# [Bis­(diphenyl­phosphino)methane-κ^2^ *P*,*P*′]dichloridopalladium(II)

**DOI:** 10.1107/S1600536808042980

**Published:** 2009-01-08

**Authors:** Muhammad Shahid, Imtiaz-ud-Din, Muhammad Mazhar, Matthias Zeller, Allen D. Hunter

**Affiliations:** a Department of Chemistry, Quaid-i-Azam University, Islamabad, 45320-Pakistan; b STaRBURSTT-Cyberdiffraction Consortium at YSU and Department of Chemistry, Youngstown State University, 1 University Plaza, Youngstown, Ohio 44555-3663, USA

## Abstract

The title complex, [PdCl_2_(C_25_H_22_P_2_)], is a slightly distorted square-planar bis­(diphenyl­phosphino)methane *cis*-complex of PdCl_2_. The structure of a polymorph of the title compound has been described earlier, but the arrangement of the mol­ecules observed in the current structure is distinctively different from that previously reported [Steffen & Palenik (1976 ▶). *Inorg. Chem.* **15**, 2432–2439]. The earlier report describes a structure with individual well separated mol­ecules crystallizing in space group *P*2_1_/*n*. The polymorph described here, which is isostructrural to its Pt analogue [Babai *et al.* (2006 ▶). *Z. Anorg. Allg. Chem.* **632**, 639–644], crystallizes in *C*2/*c* with chains of *C*2-symmetric mol­ecules stretching parallel to the *b *axis. The Pd atoms and the bis­phosphino­methane units are located on two different positions created by a non-crystallographic mirror operation with an occupancy of 0.6677 (11) for the major (PCH_2_P)Pd moiety. The positions of the Cl atoms of the minor moiety do coincide perfectly with those of the next mol­ecule along the chain parallel to *b*, and they are thus not included in the disorder. The phenyl rings also do not take part in the disorder and are common to both the major and minor moieties of the (PCH_2_P)PdCl_2_ units. Assuming no defects, mol­ecules in each chain will thus have to be oriented the same way and the effect of the disorder of the (PCH_2_P)Pd unit is thus a reversal in direction of the chains parallel to *b*. The presence of light streaks of intensity between actual Bragg peaks indicates that a somehow ordered arrangement not resolved in the Bragg diffraction data may be present (*i.e.* an incommensurate superstructure) rather than a random or domain arrangement of the chains.

## Related literature

For a different polymorph, see: Steffen & Palenik (1976 ▶). For a related structure, see: Babai *et al.* (2006 ▶). For background literature, see: Braun *et al.* (2007 ▶; Puddephatt (1983 ▶); Farina *et al.* (1997 ▶); Chaudret *et al.* (1988 ▶); Mitchell (1992 ▶); Witt & Roesky (1994 ▶); Balakrishna *et al.* (1994 ▶); Tsuji (1996 ▶); Miyaura & Suzuki (1995 ▶); Suzuki (1991 ▶); Ozawa (1997 ▶).
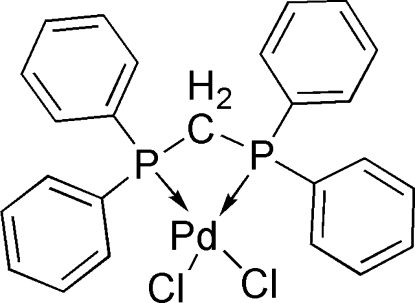


## Experimental

### 

#### Crystal data

[PdCl_2_(C_25_H_22_P_2_)]
*M* *_r_* = 561.67
Monoclinic, 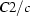

*a* = 16.137 (2) Å
*b* = 7.7836 (9) Å
*c* = 19.217 (2) Å
β = 99.029 (3)°
*V* = 2383.8 (5) Å^3^
*Z* = 4
Mo *K*α radiation
μ = 1.15 mm^−1^
*T* = 100 (2) K
0.60 × 0.40 × 0.20 mm

#### Data collection

Bruker SMART APEX CCD diffractometer
Absorption correction: multi-scan (*SADABS* in *SAINT-Plus*; Bruker, 2003 ▶) *T*_min_ = 0.635, *T*_max_ = 0.795
11903 measured reflections
2951 independent reflections
2889 reflections with *I* > 2σ(*I*)
*R*_int_ = 0.022

#### Refinement

*R*[*F* ^2^ > 2σ(*F* ^2^)] = 0.035
*wR*(*F* ^2^) = 0.078
*S* = 1.34
2951 reflections
159 parameters
H-atom parameters constrained
Δρ_max_ = 0.43 e Å^−3^
Δρ_min_ = −0.47 e Å^−3^

### 

Data collection: *SMART* (Bruker, 2002 ▶); cell refinement: *SAINT-Plus* (Bruker, 2003 ▶); data reduction: *SAINT-Plus*; program(s) used to solve structure: *SHELXTL* (Sheldrick, 2008 ▶); program(s) used to refine structure: *SHELXTL*; molecular graphics: *SHELXTL*; software used to prepare material for publication: *SHELXTL*.

## Supplementary Material

Crystal structure: contains datablocks I, global. DOI: 10.1107/S1600536808042980/pv2124sup1.cif

Structure factors: contains datablocks I. DOI: 10.1107/S1600536808042980/pv2124Isup2.hkl

Additional supplementary materials:  crystallographic information; 3D view; checkCIF report

## Figures and Tables

**Fig. 1.**
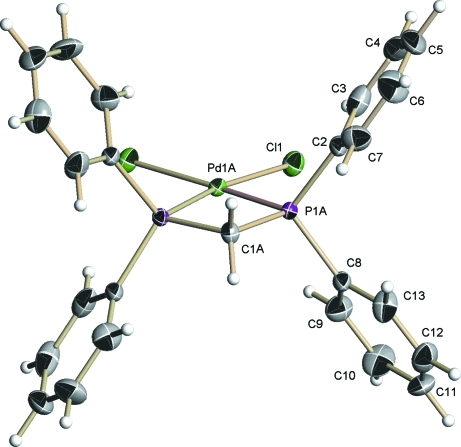
Molecular structure of the title compound showing the atom-labelling scheme and displacement ellipsoids (50% probability level); minor faction has been omitted for clarity. The un-labeled atoms are related to the labeled ones by application of the C2 axis passing through Pd1A and C1A.

**Fig. 2.**
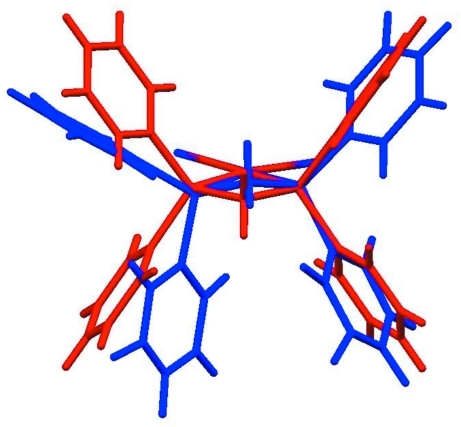
Overlay of the two polymorphs of the title compound. Red: the title compound, blue: previously reported polymorph (Steffen & Palenik, 1976).

**Fig. 3.**
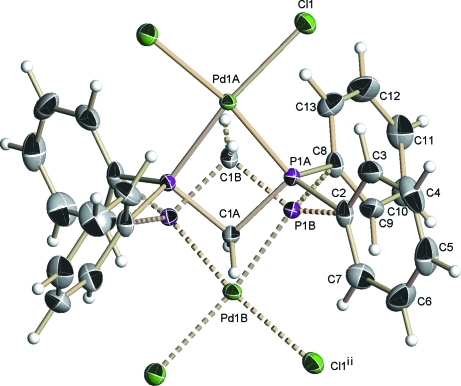
Thermal ellipsoid plot of (I) showing the disordered minor fraction of the molecule (50% probability level); dashed lines indicate bonds within the minor moiety. Symmetry operator (ii) x, y + 1, z.

**Fig. 4.**
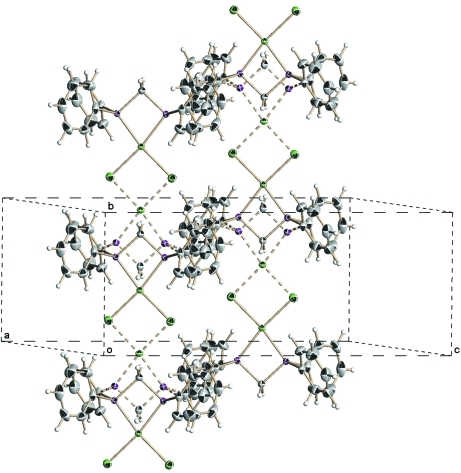
Unit cell packing diagram showing disorder within the chains parallel to the b-axis.

## supplementary crystallographic information

### Comment

Stabilization of organotransition metal complexes is commonly achieved through bidentante-chelating bisphosphine ligands of the type *X*(PPh)_2_ and these kinds of ligands were *e.g.* used to gain insight into several binuclear bond activation processes (Braun *et al.*, 2007; Puddephatt, 1983). If *X* is a one-atom-spacer such as CH_2_ (diphenylphosphinomethane or dppm) or NH (diphenylphosphinoamine or dppa), their chelate complexes result in strained 4-membered rings, and the ligands most commonly act as bridging ligands. They can, however, also act as bidentate chelating ligands and form 4-membered rings. The applications of dppm (Puddephatt, 1983; Chaudret *et al.*, 1988) and dppa (Witt & Roesky, 1994; Balakrishna *et al.*, 1994) in binuclear organometallic and coordination chemistry have been reviewed. There has been considerable interest in the properties of palladium complexes because of their frequent use as catalysts for carbon-carbon coupling reactions (Tsuji, 1996). These reactions play a key role in the synthesis of many organic chemicals, natural products and also in a variety of industrial processes. The most important examples for such a type of catalysis are the Suzuki (Miyaura & Suzuki, 1995; Suzuki, 1991) and the Stille (Farina *et al.*, 1997; Mitchell, 1992) cross-coupling reactions. Phosphine complexes of palladium (II) are well known and are prepared from palladium (II) salts with an excess of phosphine ligand quite easily (Ozawa, 1997). The complexes have been used for the establishment of catalytic activity of palladium in various reactions including CC-coupling (Tsuji, 1996).

The title compound, (I), a complex of dppm with PdCl_2_, is such a complex (Fig. 1). The complex has a slightly distorted square planar coordination environment around the Pd atom and the two chlorine atoms are, due to the restraints of the bidentate chelating bis-phosphine ligand, in *cis* position to each other. The structure of a polymorph of the title compound was described earlier, but the arrangement of the molecules observed in the current structure is distinctively different from that of previously reported (Steffen & Palenik, 1976). The earlier report describes a structure with individual well separated molecules crystallized in the monoclinic space group *P*2_1_/*n*. The molecules are arranged in a pattern typical for close packed structure, and the chlorine atoms show several weak C—H···Cl interactions with hydrogen atoms of the symmetry related molecules. The coordination environment of the Pd atom is quite asymmetric and especially the four membered PdP_2_C ring is not planar in this structure. Instead the CH_2_ unit is located above the mean plane of the Cl_2_PdP_2_ unit. The phenyl rings follow the CH_2_ group and are rotated towards each other on one side of the molecule, away from each other on the other.

The title compound is isostructrural to its Pt analogue (Babai *et al.* 2006) and crystallizes in a different space group. The molecules of (I) lie on a crystallographic two fold axis that stretches through the Pd atom and the CH_2_ group of the ligand, and the molecules are C2-symmetric (Fig. 1). The Pd atoms in (I) exhibit a much regular arrangement than in its Pt analogue and the PdCl_2_P_2_ units are essentially planar; especially the CH_2_ unit is located within the mean plane of the PdCl_2_P_2_ fragment, and the positions of the phenyl groups are the same on both sides of the PdCl_2_P_2_ plane. The pronounced differences in the geometries of the two polymorphs can be easily seen in an overlay of the molecules as shown in Figure 2. In (I), the Pd—Cl and Pd—P bond distances are 2.3933 (7) and 2.2288 (11) Å, respectively; these distances vary by 0.10 and 0.14 Å, respectively, in the other polymorph.

The individual molecules in (I) are arranged in a head to tail fashion in chains parallel to the *b*-axis of the unit cell with the Cl atoms pointing towards the P—CH_2_—P end of a symmetry related molecule. This arrangement does allow an interesting type of disorder to manifest itself. The palladium atoms and the bisphosphinomethane units are disordered over two different positions created by a non-crystallographic mirror operation. Figure 3 shows the disorder for one individual molecule. The occupancy of the major (PCH_2_P)Pd moiety refined to 0.6677 (10) (a similar disorder, but to a lesser extent, was observed in the Pt analogue). The positions of the chlorine atoms of the minor component do however coincide perfectly with those of the next molecule along the chain parallel to *b*, and they are thus not part of the disorder: the displacement parameters of the Cl atoms are essentially isotropic and the largest residual electron densities are located in the C—C bonds of the phenyl rings, thus excluding disorder of the chlorine atom positions. The phenyl rings are also not disordered and are common to both the major and minor components of the molecule with only some slightly pronounced anisotropic displacement parameters of the carbon atoms hinting towards a small adjustment of the C atom positions within the two moieties. Assuming no defect molecules in each chain will thus have to be oriented the same way and the effect of the disorder of the (PCH_2_P)Pd unit is thus a reversal in direction of the chains parallel to the *b*-axis as depicted in Figure 4.

The Pd—P distances in the minor fraction of the (I) differ only slightly compared to those in the major one (2.231 (2) *versus* 2.2288 (11) Å). The Pd—Cl distances, however, do show significant differences, at 2.3933 (7) and  2.4591 (8) Å for the major and the monor fractions, respectively. The Pd—Cl distances in the previously described polymorph are shorter than both of these values (2.362 (1) and 2.352 (1) Å, respectively). It has to be pointed out, however, that light streaks of itensity are found between the positions of the Bragg peaks, thus pointing towards the possible presence of an incommensurate superstructure rather than a random or domain arrangement.

### Experimental

(1.08 g, 2.82 mmol) dppm was added to a stirred suspension of PdCl_2_ (0.50 g, 2.82 mmol) in 20 ml of methanol in a Schlenk tube under inert atmosphere. After two hours stirring, the mixture was evaporated under vacuum to dryness and the solid was recrystallized from DMSO to obtain yellowish block-shaped crystals at room temperature after several days.

### Refinement

The molecule is flip disordered over two positions with site occupancy factors of 0.6677 (10) and 0.3323 (10). The phenyl rings for both orientations occupy approximately the same positions and have been refined as not disordered. Based on the appearance of the diffraction frames (they show streaks of intensity between the actual diffraction spots) the disorder seems to be not random but possibly an incommensurate superstructure is present. No restraints have been applied. All hydrogen atoms were placed in calculated positions with C—H distances 0.99 and 0.95 Å for methylene and aryl H-atoms, respectively, and were included in the refinements in a riding mode with isotropic displacement parameters 1.2 times that of the parent carbon atoms.

### Crystal data

**Table:**

[PdCl_2_(C_25_H_22_P_2_)]	*F*(000) = 1128
*M**_r_* = 561.67	*D*_x_ = 1.565 Mg m^−^^3^
Monoclinic, *C*2/*c*	Mo *K*α radiation, λ = 0.71073 Å
Hall symbol: -C 2yc	Cell parameters from 5783 reflections
*a* = 16.137 (2) Å	θ = 2.9–30.5°
*b* = 7.7836 (9) Å	µ = 1.15 mm^−^^1^
*c* = 19.217 (2) Å	*T* = 100 K
β = 99.029 (3)°	Block, yellow
*V* = 2383.8 (5) Å^3^	0.60 × 0.40 × 0.20 mm
*Z* = 4	

### Data collection

**Table:**

Bruker SMART APEX CCD diffractometer	2951 independent reflections
Radiation source: fine-focus sealed tube	2889 reflections with *I* > 2σ(*I*)
graphite	*R*_int_ = 0.022
ω scans	θ_max_ = 28.3°, θ_min_ = 2.2°
Absorption correction: multi-scan (*SADABS* in *SAINT-Plus*; Bruker, 2003)	*h* = −21→21
*T*_min_ = 0.635, *T*_max_ = 0.795	*k* = −10→10
11903 measured reflections	*l* = −25→25

### Refinement

**Table:**

Refinement on *F*^2^	Primary atom site location: structure-invariant direct methods
Least-squares matrix: full	Secondary atom site location: difference Fourier map
*R*[*F*^2^ > 2σ(*F*^2^)] = 0.035	Hydrogen site location: inferred from neighbouring sites
*wR*(*F*^2^) = 0.078	H-atom parameters constrained
*S* = 1.34	*w* = 1/[σ^2^(*F*_o_^2^) + (0.0001*P*)^2^ + 8.3555*P*] where *P* = (*F*_o_^2^ + 2*F*_c_^2^)/3
2951 reflections	(Δ/σ)_max_ < 0.001
159 parameters	Δρ_max_ = 0.43 e Å^−^^3^
0 restraints	Δρ_min_ = −0.46 e Å^−^^3^

### Special details

**Table:**

Geometry. All e.s.d.'s (except the e.s.d. in the dihedral angle between two l.s. planes) are estimated using the full covariance matrix. The cell e.s.d.'s are taken into account individually in the estimation of e.s.d.'s in distances, angles and torsion angles; correlations between e.s.d.'s in cell parameters are only used when they are defined by crystal symmetry. An approximate (isotropic) treatment of cell e.s.d.'s is used for estimating e.s.d.'s involving l.s. planes.
Refinement. Refinement of *F*^2^ against ALL reflections. The weighted *R*-factor *wR* and goodness of fit *S* are based on *F*^2^, conventional *R*-factors *R* are based on *F*, with *F* set to zero for negative *F*^2^. The threshold expression of *F*^2^ > σ(*F*^2^) is used only for calculating *R*-factors(gt) *etc*. and is not relevant to the choice of reflections for refinement. *R*-factors based on *F*^2^ are statistically about twice as large as those based on *F*, and *R*- factors based on ALL data will be even larger.

### Fractional atomic coordinates and isotropic or equivalent isotropic displacement parameters (Å^2^)

**Table:**

	*x*	*y*	*z*	*U*_iso_*/*U*_eq_	Occ. (<1)
Pd1A	0.0000	0.09241 (5)	0.2500	0.01440 (10)	0.6677 (10)
P1A	0.02483 (5)	−0.13693 (15)	0.18695 (4)	0.01393 (18)	0.6677 (10)
C1A	0.0000	−0.2984 (6)	0.2500	0.0183 (9)	0.6677 (10)
H1B	0.0487	−0.3709	0.2690	0.022*	0.3339 (5)
H1A	−0.0487	−0.3709	0.2310	0.022*	0.3339 (5)
Pd1B	0.0000	−0.46608 (9)	0.2500	0.0170 (2)	0.3323 (10)
P1B	0.02709 (11)	−0.2360 (3)	0.18811 (9)	0.0154 (4)	0.3323 (10)
C1B	0.0000	−0.0716 (13)	0.2500	0.0198 (19)	0.3323 (10)
H1D	−0.0483	0.0009	0.2299	0.024*	0.1661 (5)
H1C	0.0483	0.0009	0.2701	0.024*	0.1661 (5)
Cl1	−0.03931 (4)	0.30720 (8)	0.32776 (3)	0.02497 (14)	
C2	0.13277 (14)	−0.1786 (3)	0.17332 (12)	0.0224 (5)	
C3	0.18792 (18)	−0.0469 (4)	0.19471 (15)	0.0323 (6)	
H3	0.1694	0.0523	0.2166	0.039*	
C4	0.27074 (19)	−0.0603 (5)	0.18403 (18)	0.0443 (8)	
H4	0.3091	0.0299	0.1991	0.053*	
C5	0.29780 (17)	−0.2026 (5)	0.15199 (16)	0.0387 (7)	
H5	0.3546	−0.2108	0.1449	0.046*	
C6	0.2426 (2)	−0.3334 (4)	0.13011 (16)	0.0376 (7)	
H6	0.2612	−0.4316	0.1076	0.045*	
C7	0.15988 (19)	−0.3222 (4)	0.14082 (15)	0.0335 (6)	
H7	0.1218	−0.4128	0.1259	0.040*	
C8	−0.04350 (15)	−0.1806 (4)	0.10459 (12)	0.0243 (5)	
C9	−0.07168 (19)	−0.3397 (4)	0.07889 (16)	0.0347 (6)	
H9	−0.0548	−0.4404	0.1053	0.042*	
C10	−0.1238 (2)	−0.3529 (5)	0.01553 (17)	0.0453 (8)	
H10	−0.1430	−0.4626	−0.0017	0.054*	
C11	−0.14816 (19)	−0.2087 (5)	−0.02285 (15)	0.0439 (8)	
H11	−0.1841	−0.2189	−0.0668	0.053*	
C12	−0.1214 (2)	−0.0502 (5)	0.00133 (17)	0.0423 (8)	
H12	−0.1389	0.0492	−0.0257	0.051*	
C13	−0.06807 (18)	−0.0336 (4)	0.06589 (16)	0.0333 (6)	
H13	−0.0491	0.0764	0.0829	0.040*	

### Atomic displacement parameters (Å^2^)

**Table:**

	*U*^11^	*U*^22^	*U*^33^	*U*^12^	*U*^13^	*U*^23^
Pd1A	0.01479 (17)	0.01283 (17)	0.01607 (17)	0.000	0.00393 (12)	0.000
P1A	0.0138 (4)	0.0149 (5)	0.0133 (4)	0.0008 (3)	0.0030 (3)	−0.0005 (3)
C1A	0.023 (2)	0.016 (2)	0.017 (2)	0.000	0.0053 (18)	0.000
Pd1B	0.0178 (4)	0.0130 (4)	0.0215 (4)	0.000	0.0069 (3)	0.000
P1B	0.0171 (8)	0.0140 (10)	0.0154 (8)	−0.0010 (7)	0.0033 (6)	0.0000 (7)
C1B	0.021 (5)	0.023 (5)	0.017 (4)	0.000	0.006 (4)	0.000
Cl1	0.0257 (3)	0.0236 (3)	0.0275 (3)	0.0016 (2)	0.0101 (2)	−0.0020 (2)
C2	0.0150 (10)	0.0364 (14)	0.0160 (10)	−0.0019 (9)	0.0031 (8)	−0.0002 (10)
C3	0.0318 (14)	0.0289 (14)	0.0360 (14)	0.0020 (11)	0.0049 (11)	−0.0092 (11)
C4	0.0293 (15)	0.050 (2)	0.0533 (19)	−0.0197 (14)	0.0054 (13)	−0.0155 (16)
C5	0.0160 (12)	0.062 (2)	0.0385 (15)	0.0048 (13)	0.0054 (11)	0.0017 (15)
C6	0.0438 (17)	0.0317 (15)	0.0402 (16)	0.0117 (13)	0.0158 (13)	−0.0010 (12)
C7	0.0392 (15)	0.0278 (14)	0.0366 (15)	−0.0140 (12)	0.0155 (12)	−0.0067 (11)
C8	0.0153 (10)	0.0421 (15)	0.0158 (10)	−0.0017 (10)	0.0032 (8)	−0.0013 (10)
C9	0.0339 (15)	0.0296 (14)	0.0397 (16)	0.0038 (12)	0.0033 (12)	0.0052 (12)
C10	0.0454 (18)	0.049 (2)	0.0385 (17)	−0.0123 (15)	−0.0010 (14)	−0.0161 (15)
C11	0.0278 (14)	0.079 (3)	0.0225 (13)	−0.0011 (15)	−0.0025 (11)	−0.0013 (15)
C12	0.0359 (16)	0.053 (2)	0.0414 (17)	0.0204 (14)	0.0175 (13)	0.0273 (15)
C13	0.0351 (15)	0.0270 (14)	0.0426 (16)	−0.0048 (11)	0.0211 (13)	−0.0064 (12)

### Geometric parameters (Å, °)

**Table:**

Pd1A—P1A	2.2288 (11)	C2—C3	1.377 (4)
Pd1A—P1A^i^	2.2288 (11)	C2—C7	1.384 (4)
Pd1A—Cl1^i^	2.3933 (7)	C3—C4	1.388 (4)
Pd1A—Cl1	2.3933 (7)	C3—H3	0.9500
P1A—C8	1.814 (3)	C4—C5	1.372 (5)
P1A—C2	1.831 (2)	C4—H4	0.9500
P1A—C1A	1.833 (3)	C5—C6	1.374 (5)
P1A—P1A^i^	2.6690 (17)	C5—H5	0.9500
C1A—P1A^i^	1.833 (3)	C6—C7	1.385 (4)
C1A—H1B	0.9900	C6—H6	0.9500
C1A—H1A	0.9900	C7—H7	0.9500
Pd1B—P1B^i^	2.231 (2)	C8—C9	1.383 (4)
Pd1B—P1B	2.231 (2)	C8—C13	1.388 (4)
Pd1B—Cl1^ii^	2.4591 (8)	C9—C10	1.371 (4)
Pd1B—Cl1^iii^	2.4591 (8)	C9—H9	0.9500
P1B—C2	1.828 (3)	C10—C11	1.367 (5)
P1B—C1B	1.846 (8)	C10—H10	0.9500
P1B—C8	1.867 (3)	C11—C12	1.364 (5)
P1B—P1B^i^	2.660 (3)	C11—H11	0.9500
C1B—P1B^i^	1.846 (8)	C12—C13	1.400 (5)
C1B—H1D	0.9900	C12—H12	0.9500
C1B—H1C	0.9900	C13—H13	0.9500
Cl1—Pd1B^iv^	2.4591 (8)		
			
P1A—Pd1A—P1A^i^	73.56 (5)	H1D—C1B—H1C	110.6
P1A—Pd1A—Cl1^i^	97.60 (3)	C3—C2—C7	120.0 (2)
P1A^i^—Pd1A—Cl1^i^	170.67 (3)	C3—C2—P1B	135.2 (2)
P1A—Pd1A—Cl1	170.67 (3)	C7—C2—P1B	104.0 (2)
P1A^i^—Pd1A—Cl1	97.60 (3)	C3—C2—P1A	114.2 (2)
Cl1^i^—Pd1A—Cl1	91.38 (3)	C7—C2—P1A	125.7 (2)
C8—P1A—C2	107.67 (11)	C2—C3—C4	119.5 (3)
C8—P1A—C1A	106.40 (12)	C2—C3—H3	120.3
C2—P1A—C1A	106.54 (11)	C4—C3—H3	120.3
C8—P1A—Pd1A	119.22 (10)	C5—C4—C3	120.6 (3)
C2—P1A—Pd1A	118.43 (9)	C5—C4—H4	119.7
C1A—P1A—Pd1A	96.50 (12)	C3—C4—H4	119.7
C8—P1A—P1A^i^	124.36 (9)	C4—C5—C6	119.9 (3)
C2—P1A—P1A^i^	123.91 (9)	C4—C5—H5	120.0
Pd1A—P1A—P1A^i^	53.22 (3)	C6—C5—H5	120.0
P1A—C1A—P1A^i^	93.4 (2)	C5—C6—C7	120.1 (3)
P1A—C1A—H1B	113.0	C5—C6—H6	119.9
P1A^i^—C1A—H1B	113.0	C7—C6—H6	119.9
P1A—C1A—H1A	113.0	C2—C7—C6	119.9 (3)
P1A^i^—C1A—H1A	113.0	C2—C7—H7	120.1
H1B—C1A—H1A	110.4	C6—C7—H7	120.1
P1B^i^—Pd1B—P1B	73.19 (11)	C9—C8—C13	119.8 (2)
P1B^i^—Pd1B—Cl1^ii^	172.28 (6)	C9—C8—P1A	126.9 (2)
P1B—Pd1B—Cl1^ii^	99.29 (5)	C13—C8—P1A	113.3 (2)
P1B^i^—Pd1B—Cl1^iii^	99.28 (5)	C9—C8—P1B	102.9 (2)
P1B—Pd1B—Cl1^iii^	172.28 (6)	C13—C8—P1B	137.3 (2)
Cl1^ii^—Pd1B—Cl1^iii^	88.28 (4)	C10—C9—C8	120.4 (3)
C2—P1B—C1B	104.60 (19)	C10—C9—H9	119.8
C2—P1B—C8	105.56 (14)	C8—C9—H9	119.8
C1B—P1B—C8	102.70 (18)	C11—C10—C9	120.1 (3)
C2—P1B—Pd1B	123.00 (13)	C11—C10—H10	119.9
C1B—P1B—Pd1B	97.3 (2)	C9—C10—H10	119.9
C8—P1B—Pd1B	119.96 (13)	C12—C11—C10	120.7 (3)
C2—P1B—P1B^i^	125.79 (13)	C12—C11—H11	119.7
C8—P1B—P1B^i^	121.81 (13)	C10—C11—H11	119.7
Pd1B—P1B—P1B^i^	53.40 (5)	C11—C12—C13	120.2 (3)
P1B—C1B—P1B^i^	92.2 (5)	C11—C12—H12	119.9
P1B—C1B—H1D	113.3	C13—C12—H12	119.9
P1B^i^—C1B—H1D	113.3	C8—C13—C12	118.8 (3)
P1B—C1B—H1C	113.3	C8—C13—H13	120.6
P1B^i^—C1B—H1C	113.3	C12—C13—H13	120.6
			
P1A^i^—Pd1A—P1A—C8	112.91 (11)	P1A^i^—P1A—C2—P1B	81.03 (16)
Cl1^i^—Pd1A—P1A—C8	−70.13 (10)	C7—C2—C3—C4	−0.6 (4)
P1A^i^—Pd1A—P1A—C2	−112.79 (11)	P1B—C2—C3—C4	167.8 (3)
Cl1^i^—Pd1A—P1A—C2	64.17 (10)	P1A—C2—C3—C4	−177.0 (2)
P1A^i^—Pd1A—P1A—C1A	0.003 (1)	C2—C3—C4—C5	0.5 (5)
Cl1^i^—Pd1A—P1A—C1A	176.96 (3)	C3—C4—C5—C6	0.1 (5)
Cl1^i^—Pd1A—P1A—P1A^i^	176.96 (3)	C4—C5—C6—C7	−0.4 (5)
C8—P1A—C1A—P1A^i^	−123.07 (10)	C3—C2—C7—C6	0.3 (4)
C2—P1A—C1A—P1A^i^	122.25 (10)	P1B—C2—C7—C6	−171.4 (3)
Pd1A—P1A—C1A—P1A^i^	0.0	P1A—C2—C7—C6	176.2 (2)
P1B^i^—Pd1B—P1B—C2	112.72 (16)	C5—C6—C7—C2	0.3 (5)
Cl1^ii^—Pd1B—P1B—C2	−65.50 (14)	C2—P1A—C8—C9	80.5 (3)
P1B^i^—Pd1B—P1B—C1B	−0.002 (1)	C1A—P1A—C8—C9	−33.4 (3)
Cl1^ii^—Pd1B—P1B—C1B	−178.23 (6)	Pd1A—P1A—C8—C9	−140.8 (2)
P1B^i^—Pd1B—P1B—C8	−109.25 (16)	P1A^i^—P1A—C8—C9	−77.5 (3)
Cl1^ii^—Pd1B—P1B—C8	72.53 (14)	C2—P1A—C8—C13	−99.7 (2)
Cl1^ii^—Pd1B—P1B—P1B^i^	−178.22 (6)	C1A—P1A—C8—C13	146.4 (2)
C2—P1B—C1B—P1B^i^	−126.92 (15)	Pd1A—P1A—C8—C13	39.0 (2)
C8—P1B—C1B—P1B^i^	123.03 (14)	P1A^i^—P1A—C8—C13	102.30 (18)
Pd1B—P1B—C1B—P1B^i^	0.0	C2—P1A—C8—P1B	74.12 (18)
P1A^i^—Pd1A—Cl1—Pd1B^iv^	177.46 (3)	C1A—P1A—C8—P1B	−39.78 (18)
Cl1^i^—Pd1A—Cl1—Pd1B^iv^	0.0	Pd1A—P1A—C8—P1B	−147.23 (17)
C1B—P1B—C2—C3	−3.2 (4)	P1A^i^—P1A—C8—P1B	−83.88 (16)
C8—P1B—C2—C3	104.8 (3)	C2—P1B—C8—C9	112.9 (2)
Pd1B—P1B—C2—C3	−112.2 (3)	C1B—P1B—C8—C9	−137.7 (3)
P1B^i^—P1B—C2—C3	−46.3 (3)	Pd1B—P1B—C8—C9	−31.5 (2)
C1B—P1B—C2—C7	166.5 (3)	P1B^i^—P1B—C8—C9	−94.58 (19)
C8—P1B—C2—C7	−85.5 (2)	C2—P1B—C8—C13	−64.0 (3)
Pd1B—P1B—C2—C7	57.5 (2)	C1B—P1B—C8—C13	45.4 (4)
P1B^i^—P1B—C2—C7	123.43 (18)	Pd1B—P1B—C8—C13	151.6 (3)
C1B—P1B—C2—P1A	−38.5 (2)	P1B^i^—P1B—C8—C13	88.5 (3)
C8—P1B—C2—P1A	69.47 (18)	C2—P1B—C8—P1A	−72.34 (18)
Pd1B—P1B—C2—P1A	−147.5 (2)	C1B—P1B—C8—P1A	37.0 (2)
P1B^i^—P1B—C2—P1A	−81.59 (15)	Pd1B—P1B—C8—P1A	143.3 (2)
C8—P1A—C2—C3	129.4 (2)	P1B^i^—P1B—C8—P1A	80.15 (15)
C1A—P1A—C2—C3	−116.8 (2)	C13—C8—C9—C10	−0.1 (4)
Pd1A—P1A—C2—C3	−9.6 (2)	P1A—C8—C9—C10	179.7 (2)
P1A^i^—P1A—C2—C3	−72.5 (2)	P1B—C8—C9—C10	−177.7 (3)
C8—P1A—C2—C7	−46.8 (3)	C8—C9—C10—C11	0.1 (5)
C1A—P1A—C2—C7	67.1 (3)	C9—C10—C11—C12	−0.2 (5)
Pd1A—P1A—C2—C7	174.2 (2)	C10—C11—C12—C13	0.3 (5)
P1A^i^—P1A—C2—C7	111.4 (2)	C9—C8—C13—C12	0.1 (4)
C8—P1A—C2—P1B	−77.09 (18)	P1A—C8—C13—C12	−179.7 (2)
C1A—P1A—C2—P1B	36.71 (18)	P1B—C8—C13—C12	176.6 (2)
Pd1A—P1A—C2—P1B	143.87 (17)	C11—C12—C13—C8	−0.2 (4)

Symmetry codes: (i) −*x*, *y*, −*z*+1/2; (ii) −*x*, *y*−1, −*z*+1/2; (iii) *x*, *y*−1, *z*; (iv) *x*, *y*+1, *z*.
